# Measuring Personality through Images: Validating a Forced-Choice Image-Based Assessment of the Big Five Personality Traits

**DOI:** 10.3390/jintelligence10010012

**Published:** 2022-02-07

**Authors:** Airlie Hilliard, Emre Kazim, Theodoros Bitsakis, Franziska Leutner

**Affiliations:** 1 Institute of Management Studies, Goldsmiths, University of London, New Cross, London SE14 6NW, UK; f.leutner@gold.ac.uk; 2 Holistic AI, 18 Soho Square, London W1D 3QH, UK; e.kazim@ucl.ac.uk; 3 Department of Computer Science, University College London, Gower St., London WC1E 6EA, UK; 4 HireVue, Riverbank House, 2 Swan Lane, London EC4R 3TT, UK; tbitsakis@hirevue.com

**Keywords:** personality, Big Five, image-based measure, psychological assessment, machine learning, bias

## Abstract

Selection methods are commonly used in talent acquisition to predict future job performance and to find the best candidates, but questionnaire-based assessments can be lengthy and lead to candidate fatigue and poor engagement, affecting completion rates and producing poor data. Gamification can mitigate some of these issues through greater engagement and shorter testing times. One avenue of gamification is image-based tests. Although such assessments are starting to gain traction in personnel selection, few studies describing their validity and psychometric properties exist. The current study explores the potential of a five-minute, forced-choice, image-based assessment of the Big Five personality traits to be used in selection. Study 1 describes the creation of the image pairs and the selection of the 150 best-performing items based on a sample of 300 respondents. Study 2 describes the creation of machine-learning-based scoring algorithms and tests of their convergent and discriminate validity and adverse impact based on a sample of 431 respondents. All models showed good levels of convergent validity with the IPIP-NEO-120 (openness *r* = 0.71, conscientiousness *r* = 0.70, extraversion *r* = 0.78, agreeableness *r* = 0.60, and emotional stability *r* = 0.70) and were largely free from potential adverse impact. The implications for recruitment policy and practice and the need for further validation are discussed.

## 1. Introduction

This article explores the potential for measuring the personality of job applicants through their image choices using a novel measure whereby respondents are asked to indicate which image in a pair is more like them. We report on two studies, where the first describes the creation and refinement of the measure and the second creates scoring algorithms and validates them by assessing convergent and discriminant validity and the potential for adverse impact. Although some image-based personality assessments exist commercially (e.g., Traitify and RedBull Wingfinder), there is little literature in this field. Since applicant perceptions of the selection process can influence whether an applicant is likely to accept a job offer (Hausknecht et al. 2004), applicant experience is pertinent for both applicants and hiring teams. Image-based formats might increase engagement and thereby applicant perception, as they increase satisfaction and elicit stronger responses compared with questionnaire-based measures (Downes-Le Guin et al. 2012; Meissner and Rothermund 2015). 

The purpose of this article is to address this lack of validation in the research regarding image-based assessments of personality, particularly those created for use in selection. Our findings provide preliminary evidence that assessments of this type could be a valid and fairer alternative to questionnaire-based selection assessments that use Likert scales. 

The article begins with a discussion about selection assessments, particularly those measuring cognitive ability or personality, followed by evidence in favor of the use of game- and image-based assessments, such as their shorter testing times (Atkins et al. 2014; Leutner et al. 2020). Before we describe the method used in each study and evaluate the performance of the assessment, we outline the need for selection assessments, focusing on those measuring cognitive ability and personality. Specifically, we compare the fairness of measures of these constructs and the scope for assessing them through game- and image-based assessments. As will be highlighted below, much of the research into gamification has focused on cognitive ability, but there is evidence that image choices can be used to measure personality (Krainikovsky et al. 2019; Leutner et al. 2017); notwithstanding this, a validated Big Five personality measure created for use in selection has not been described in peer-reviewed research. The reported study, therefore, aims to contribute towards the lack of evidence addressing the potential for soft skills, such as personality, to be measured through gamified assessments, particularly those using an image-based format. We do so through an integrated approach, drawing from psychology and machine learning to create and validate the measure. This feasibility study found that all five traits can be accurately measured through an image-based format, with convergent validity similar to that between other traditional measures of personality. While we note that further investigation is needed into the assessment and in particular its lower discriminant validity compared to questionnaire-based measures, our preliminary findings demonstrate that a forced-choice, image-based assessment has the potential to be a valid way of measuring the personality of applicants following further validation. We discuss the implications for recruitment policy and practice and the need for further validation.

### 1.1. Assessment in Selection

Selection methods have been used in recruitment for over 100 years to evaluate candidate suitability and predict future job performance (Ryan and Ployhart 2013; Schmidt et al. 2016; Schmidt and Hunter 1998), with around 40 million assessments being completed globally by candidates each year (Chamorro-Premuzic 2017). The most valid predictor of job performance is cognitive ability (Schmitt 2014), with validity estimates of *r* = 0.51 (Schmidt and Hunter 1998), a value that increases when combined with integrity tests (*r* = 0.65), work sample tests (*r* = 0.63), structured interviews (*r* = 0.63), or tests of conscientiousness (*r* = 0.60) (Schmidt and Hunter 1998). When used alone, the Big Five personality traits—openness to experience, conscientiousness, extraversion, agreeableness, and emotional stability—are also predictive of job performance. While conscientiousness is the most valid trait for predicting personality across multiple job contexts (Barrick and Mount 1991; Higgins et al. 2007; Judge et al. 1999; Kuncel et al. 2010; Schmitt 2014), when predicting performance for jobs that require specific skills, other personality traits are also useful (Kuncel et al. 2010). For example, extraversion predicts the job and training proficiency of those with sales and managerial jobs while openness and extraversion both predict training proficiency across occupations including police officers, sales staff, professionals, managers, and skilled/semi-skilled workers (Barrick and Mount 1991). For those working in pharmaceutics, openness, extraversion, and conscientiousness predict task performance, while agreeableness and openness predict creativity (Rothmann and Coetzer 2003). The strongest combination is the measures of cognitive ability and personality, which demonstrate incremental validity over each other and predict distinct areas of performance (Leutner and Chamorro-Premuzic 2018). However, assessments of cognitive ability are often associated with adverse impact (Hausdorf et al. 2003), describing differences in selection rates between different groups (De Corte et al. 2007). Personality assessments, on the other hand, have little to no adverse impact so are a fairer way of assessing candidates while still being predictive of job performance (Hogan et al. 1996).

### 1.2. Game and Image-Based Assessments 

While personality is often assessed via self-report methods using Likert scales, a recent trend in selection is gamification (Chamorro-Premuzic et al. 2017; Winsborough and Chamorro-Premuzic 2016) where elements of a game are added to traditional assessments to increase engagement (Armstrong et al. 2016a), including progress bars and visual and audio feedback (Landers et al. 2021). Common issues with self-reported assessments are poor response quality (Krosnick 1991) and lack of completion (Yan et al. 2011) due to the lengthy nature of scales, resulting in poor data. Game-based assessments (GBAs) can overcome these issues by offering a more engaging experience for participants (Lieberoth 2015) and shorter testing times (Atkins et al. 2014; Leutner et al. 2020), resulting in them being viewed more favorably by test-takers compared to traditional assessments (Georgiou and Nikolaou 2020; Leutner et al. 2020). GBAs also elicit less test-taking anxiety (Mavridis and Tsiatsos 2017; Smits and Charlier 2011), are less prone to social desirability bias than questionnaire-based measures, and can be more predictive of future job performance if they are designed to measure job-related behaviors (Armstrong et al. 2016b). The algorithms created for GBAs are often tested for and mitigate against adverse impact (HireVue 2019; Pymetrics 2021), therefore reducing the potential for the bias associated with traditional assessments (Hausdorf et al. 2003). 

To date, much of the focus of research into gamification has been on using games to assess cognitive ability (e.g., Quiroga et al. 2015, 2016), with less of a focus on measurements of soft skills such as personality. However, scores on the Big Five traits have been predicted based on image preferences with convergent validity with the NEO-PI-R (Costa and McCrae 2008) ranging from *r* = 0.06 for neuroticism to *r* = 0.28 for agreeableness (Krainikovsky et al. 2019). In their study, Krainikovsky and colleagues (2019) predicted scores using algorithms based on image preferences from a selection of images tagged with information relating to objects, behavior, emotions, and scenery, although the measure was not fully validated or created for use in selection. An image-based format further reinforces the benefits of game-based assessments and has the additional advantage of being language-neutral, meaning they can easily be adapted for use in other languages (Paunonen et al. 1990) unlike questionnaire-based measures, which often need to be redeveloped in the target language (Zhang et al. 2017). Language neutrality also has the benefit of increasing the accessibility of the assessment to those with low literacy or those with a learning difference such as dyslexia, reducing the barriers that may prevent them from securing employment (De Beer et al. 2014) if their underlying ability is obscured by language difficulties which cause them to perform poorly on verbal assessments. 

While there is a lack of validated image-based measures of all five personality traits, particularly those for use in selection, Leutner and colleagues’ (2017) validated image-based measure of creativity accurately measured openness to experience. Their measure, created for use in selection, presented respondents with images and asked them to indicate which was most like them. Creativity scores on traditional measures were accurately predicted, with good concurrent validity with the traditional scales for all three creativity constructs, namely curiosity (*r* = 0.35), cognitive flexibility (*r* = 0.50) and openness to experience (*r* = 0.50). To build on these findings, the reported study describes an image-based assessment of all five personality traits with a similar format to that of Leutner and colleagues’ (2017) creativity measure, with respondents being asked to indicate which image in a pair is more like them. The scope for developing an image-based assessment of personality for use in selection is explored through the creation of machine-learning-based predictive scoring algorithms and testing adverse impact and convergent validity between the image-based assessment and the questionnaire-based IPIP-NEO-120 (Johnson 2014). Based on the findings that image preferences can be used to predict personality (Krainikovsky et al. 2019) and that openness to experience can be predicted through image choices (Leutner et al. 2017), we expected that our preliminary findings would indicate that there is potential for all five traits to be measured through image choices.

## 2. Materials and Methods

In this section, we outline the methods used in the two studies summarized below. We draw upon methods from industrial/organizational psychology and machine learning to develop the measure and create and validate the machine-learning-based scoring algorithms used by the assessment. Overall, the aim these feasibility studies is to explore the potential for creating a valid image-based measure of personality to be used in talent recruitment in the future. An image-based format was chosen as previous findings have indicated personality can be measured through image choices (Leutner et al. 2017; Krainikovsky et al. 2019). Two studies were conducted:

- Study 1—Item Bank Creation: Study 1 describes the creation of an item pool of image pairs, along with the selection of the 150 best-performing items (image pairs), and the mapping of these items to the Big Five traits;
- Study 2—Measure Validation: Study 2 describes the development of predictive machine-learning-based scoring algorithms based on a panel of respondents. This approach, where algorithms are developed that predict outcomes on traditional assessments, is common practice in predictive measures of personality (e.g., Bachrach et al. 2012; Kosinski et al. 2013; Leutner et al. 2017; Schwartz et al. 2013), as they convert binary choices to a more interpretable output that is more reflective of the continuous nature of the Big Five traits. Study 2 also describes the validation of the assessment through measuring convergent and discriminant validity with Johnson’s (2014) questionnaire-based IPIP-NEO-120 and tests for potential adverse impact.

**Figure 1:**
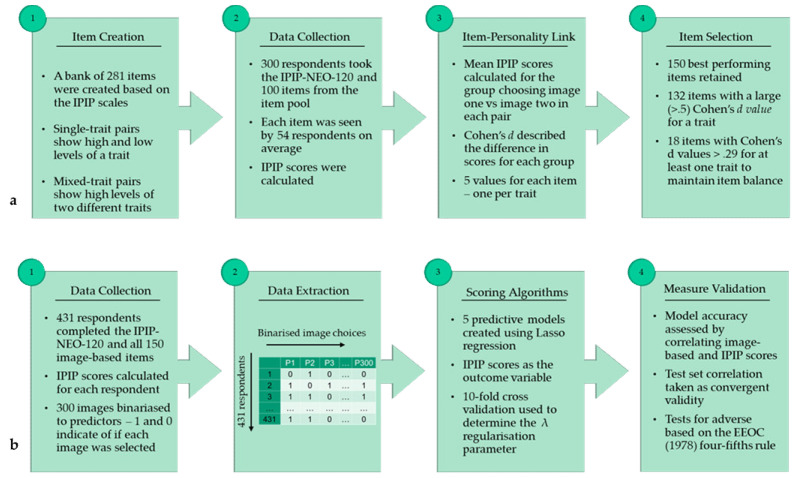
(**a**) Study 1 overview: Item creation and selection of the best-performing items for the image-based Big Five measure. (**b**) Study 2 overview: Creation of scoring algorithms and tests of convergent validity with the questionnaire-based measure and adverse impact.

An overview of the studies, which we expand upon below, can be seen in Figure 1.

### 2.1. Study 1: Item Bank Creation

#### 2.1.1. Image-Based Measure 

**Figure 2:**
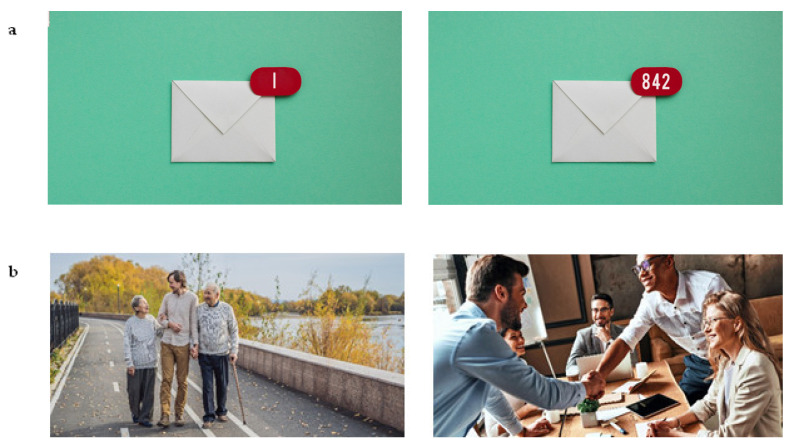
Examples of single-trait pairs. (**a**) is designed to measure the ““I like to tidy up” statement from the orderliness facet of conscientiousness. (**b**) is designed to measure the “I look at the bright side of life” statement from the cheerfulness facet of extraversion.

To create the image pairs, statements from Goldberg’s (1992) IPIP scales were used to guide image searches and ensure a good representation of the traits. Based on these statements, a team of I-O psychologists brainstormed how they could be represented through images. For example, for the statement “I like to tidy up”, the concept was a messy versus tidy email inbox (Figure 2). Images were sourced from Shutterstock due to the wide range of high-quality images available. The image database was searched using keywords related to the conceptualisation of the statements (e.g., searching for ‘email notification’). Image pairs, or items, were designed to represent multiple ethnicities, age groups, and genders and the facets of neuroticism associated with mental health (anxiety and depression) were not included in the measure. Although it could be argued that the removal of these facets could alter the structure of the emotional stability construct, this action was taken to prevent discrimination against respondents with mental health issues, particularly since the measure is forced-choice, meaning this could be interpreted as asking respondents whether they have a mental health condition or not. Caution was also taken to ensure that the images would be suitable for professional use, with scenes featuring alcohol, parties, and inappropriately dressed models being avoided. Some images were edited to remove unnecessary text, which would have prevented language neutrality, or to allow the image to be cropped more effectively. 

**Figure 3:**
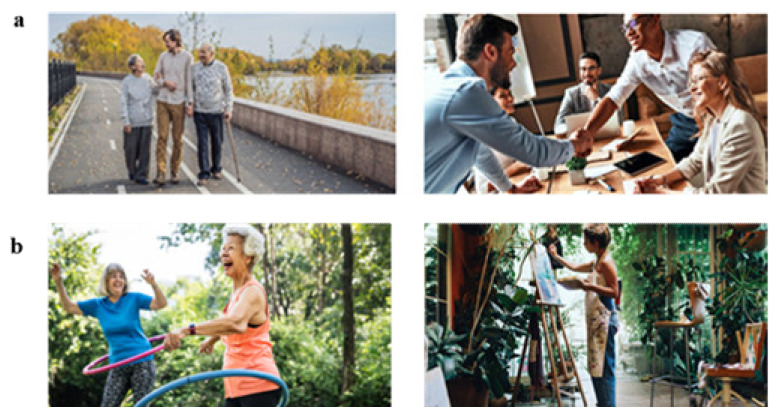
Examples of mixed-trait image pairs. (**a**) is designed to map onto the “I love to help others” statement from the altruism facet of agreeableness (**left**) and the “I feel comfortable around others” statement from the friendliness facet of extraversion (**right**). (**b**) is designed to be mapped onto the “I act comfortably around others” statement from the friendliness facet of extraversion (**left**) and “I believe in the importance of art” statement from the artistic interests facet of openness (**right**).

The image pairs were either designed to be single-trait, where the images represent high and low levels of the trait, or mixed-trait, where the images reflect high levels of two different traits to determine which trait the respondent identifies with most, with some of these pairs being presented with adjectives to aid understanding. An example of each type of image pair can be seen in Figure 2 and Figure 3. Once the images had been processed (edited and cropped), they were uploaded to the game development platform for the developers to create a functional assessment. The measure is completed on a smartphone device, with image pairs being presented one at a time along with the statement “I am more like…”, prompting respondents to select the image they identify with most in the pair, thus being forced-choice. Audio and visual feedback was added to gamify the measure (Landers et al. 2021), including a progress bar at the top and sound effects when an image was selected, as well as a pause button to allow respondents to pause and resume the assessment. 

#### 2.1.2. Questionnaire-Based Measure

The IPIP-NEO-120 (Johnson 2014) measures each trait through 24 questions using a five-point Likert scale, with a maximum score of 120 for each trait. Each trait is divided into six facets, with four questions measuring each, e.g., the cheerfulness facet of extraversion is measured by statements like “I radiate joy” and “I love life.” Items measuring neuroticism were reversed to reflect emotional stability.

#### 2.1.3. Participants

Three hundred compensated respondents were recruited through the online participant pool Prolific Academic (*M_age_* = 31.14, *SD* = 9.26, 69% female). Respondents completed the questionnaire-based measure along with 100 items from the image-based measure to avoid test-taking fatigue, resulting in each item being completed by an average of 54 participants (95% CI: 38–68). 

#### 2.1.4. Item Selection 

To select the best-performing items and reduce the length of the assessment, Cohen’s *d* values were used to describe the difference in mean IPIP scores for the group of respondents choosing image one versus image two in each pair. This was calculated for each trait. Items that had large Cohen’s *d* values, indicating a large difference in personality scores between those selecting image one versus image two, were considered to perform well. Based on these values, 150 items, or 300 images, were selected to be retained: 132 items with moderate to large values (>0.5 for a trait), and 18 items that showed small to moderate differences (>0.29 on at least one trait) to maintain a balance in the items for each trait. Using the Cohen’s *d* values, items were mapped to the trait that corresponded to the highest value. As can be seen in Table S1, of the 300 images that were retained, almost two thirds (60%) of them were mapped onto the trait that they were designed to measure. The 150 items included in the assessment had a mean highest Cohen’s *d* value of 0.77 (*SD* = 0.25; range: 0.29–1.77).

### 2.2. Study 2: Measure Validation

#### 2.2.1. Participants 

A second sample of 431 compensated respondents were recruited using Prolific Academic. Respondents completed the IPIP-NEO-120 and the full 150-item image-based assessment from study 1. The majority (*n* = 222) of respondents were female and most (*n* = 356) were under the age of 40. 209 were White, 73 Black, 66 Asian, 56 Hispanic, and 14 were of Mixed Race. 

#### 2.2.2. Analysis

A separate scoring algorithm was created for each of the five traits using a machine-learning-based predictive model with scores for the relevant trait on the questionnaire-based measure as the outcome variable. The predictors, created by binarizing the 300 images to indicate whether they were chosen by each respondent, were entered into a least absolute shrinkage and selection operator (Lasso; Tibshirani 1996) regression to create the models based on a training portion (70%) of the data. Lasso regression was favored over ordinary least squares (OLS) regression, which is commonly used in behavioral sciences, as it is prone to overfitting and inflating R^2^ values, leading to overfitting and consequently a lack of generalizability due to variance between datasets (for an exposition of the Lasso method see McNeish 2015). Lasso regression reduces the effects of variance by adding some bias to the model and introduces a regularization parameter, known as λ, which decreases the size of all of the coefficients by an equal amount. As a result of λ, some coefficients are reduced to zero (McNeish 2015) and removed from the model, creating a more interpretable model with fewer variables (Tibshirani 1996). The removal of predictors also enabled investigation of whether there was crossover in the predictors retained by each model, as well as whether only image pairs mapped to that trait were predictive (See Table S1 for trait mapping and predictor retention). To determine the most appropriate hyperparameters for the models, 10-fold cross validation was used. The remaining 30% of the data acted as an unseen sample, allowing the generalizability of the models beyond the training dataset to be examined (Jacobucci et al. 2016). The scores predicted by the model were correlated with the scores on the IPIP to determine the model’s accuracy (Cui and Gong 2018), with the correlations for the test set also being used to determine convergent validity. 

The potential for adverse impact was determined for age, gender, and ethnicity using the four-fifths rule, the two standard deviations rule, and Cohen’s *d* effect sizes. Group differences in scores were examined based on a pass or fail criteria determined by whether a respondent scored above or below the average score for that trait. According to the four-fifths rule, if the pass rate of a group is less than four-fifths of the pass rate of the group with the greatest pass rate, adverse impact is occurring (Equal Employment Opportunity Commission; EEOC et al. 1978). According to the two standard deviations rule, also known as the Z-test (Morris and Lobsenz 2000), if the disparity between the expected and observed pass rates are greater than two standard deviations, adverse impact is occurring (Office of Federal Contract Compliance Programs 1993). Finally, Cohen’s *d* can be used to determine the effect size of the difference between the mean scores of two groups, with *d* = +/−.20 indicating a small effect size, *d* = +/−.50 indicating a medium effect size, and *d* = +/−.80 indicating a large effect size (Cohen 1992). All three types of analysis were used to more robustly test for group differences since the sample size is relatively small. However, group differences in scores are not always indicative of adverse impact and could instead reflect genuine group differences in ability (Society for Industrial and Organizational Psychology 2018).

## 3. Results

In this section, we evaluate the performance of the scoring algorithms created in study 2. We first present descriptive statistics for both the questionnaire-based measure and the novel image-based measure and subsequently present the metrics used to determine the performance of the models. We assess the convergent and discriminant validity between the questionnaire and image-based measures and test for potential adverse impact. 

### 3.1. Descriptive Statistics

The descriptive statistics for scores on the IPIP-NEO-120 and image-based measure can be seen in Table 1. While the skewness and kurtosis values for these scores indicate that there may be a slight divergence from a normal distribution, the values are below the thresholds (two for skewness and seven for kurtosis) to be considered as substantially deviating from a normal distribution (Kim 2013; West et al. 1995). Internal consistency of the questionnaire-based measure, determined by Cronbach’s alpha (Cronbach 1951), was high, ranging from 0.83 for openness to experience to 0.93 for emotional stability (see Table 2). This range is consistent with that reported by Johnson (2014), which ranged from 0.83 for openness to experience to 0.90 for emotional stability. The descriptive statistics for both measures are similar, suggesting that there is a similar distribution of scores.

Although the Big Five traits are five different constructs, they intercorrelate (Chang et al. 2012). The intercorrelations for scores on the questionnaire-based measure, as seen in Table 2, concurred with intercorrelations that would usually be reported, ranging from 0.09 between openness to experience and emotional stability to 0.61 between conscientiousness and emotional stability. One reason for the high level of intercorrelation between emotional stability and conscientiousness could be because of the removal of some facets from emotional stability, which would leave the sub-scales for emotional stability that might be closer to conscientiousness (less neurotic behaviors). 

### 3.2. Model Performance

The number of predictors retained in the models ranged from 13 for openness to 32 for extraversion (see Table S2 for coefficients and mapping of the predictors retained by each model), suggesting that personality could be measured through shorter assessments and that they can offer similar insights into personality as longer assessments. Indeed, only 68 of the 150 items were retained across the five models, suggesting that the Big Five can be rapidly measured through a small number of images in around two minutes. The performance for each of the scoring algorithms created for the image-based assessment can be seen in Table 3, where correlations between scores on the image- and questionnaire-based assessments indicate model accuracy (Cui and Gong 2018). While correlations for all models were stronger for the training set, the test set correlations remained strong and significant, suggesting that the models can be generalized to unseen datasets (Jacobucci et al. 2016). This is important when creating a scoring algorithm since the models will be applied to datasets other than the ones they were trained on.

The test set correlations were also used to assess convergent validity, with convergence ranging from 0.60 for agreeableness to 0.78 for extraversion, indicating that the image-based format can be used to measure personality in a similar way to traditional, questionnaire-based formats. To better assess the convergent and discriminant correlations of the image- and questionnaire-based methods, a multitrait-multimethod approach was used (Campbell and Fiske 1959). As can be seen in Table 4, in the majority of cases, the discriminant correlations were smaller than the convergent. While for emotional stability in particular, the discriminate correlations were relatively high, the same pattern is seen in Table 2. This result could also be explained by the removal of the anxiety and depression facets from emotional stability since the remaining facets are closer to those of other traits, such as conscientiousness.

### 3.3. Adverse Impact Analysis

The results of the adverse impact analysis for the image-based assessment can be seen in Table 5. Adverse impact ratios below 0.80, medium to large effect sizes, and cases greater than +/−2 standard deviations indicated that there were group differences. Group differences were considered to be present when at least two of the metrics were in agreement. Based on these measures, potential for adverse impact was found against male and Asian respondents for the openness model, against Hispanic respondents for the conscientiousness model, and against males, Asians, and Hispanics for the agreeableness model. 

To examine whether these group differences resulted from the scoring algorithms or scores on the questionnaire-based measure, adverse impact analysis was also conducted for the IPIP-NEO-120. As can be seen in Table 6, the group differences found for the image-based assessment echo those of the questionnaire-based assessment, suggesting that the group differences identified in the image-based measure were due to group differences in scores on the questionnaire-based measure and not due to the image-based format. This highlights the need to examine group differences in the training data, since machine learning algorithms can amplify this bias (Mehrabi et al. 2021). The group differences may be due to measurement bias in the questionnaire-based assessment or could reflect genuine differences in ability since group differences are not always indicative of bias (Society for Industrial and Organizational Psychology 2018).

Since group differences were observed for the questionnaire-based measure, measurement bias was investigated by examining whether convergence varied by subgroup (Tay et al. 2021). As can be seen in Table 7, there are differences in the convergence for subgroups, and these differences echo the group differences in scores for both the image- and questionnaire-based measures. For example, the convergence for Black and Asian respondents for agreeableness is significantly lower than that of White and Hispanic respondents, with group differences being found in their scores.

## 4. Discussion

In this section, we discuss the performance of the scoring algorithms created for the reported image-based assessment of personality and the possible limitations that could result from the relatively small sample used in this study. Specifically, we discuss the performance of the models and methodological considerations. We also suggest some areas for further research before this assessment can be deployed in practice, and the implications that our preliminary findings may have for the use of image-based measures of personality in selection.

This study aimed to create scoring algorithms for and to validate a novel, image-based measure of the Big Five personality traits to explore the potential for such a measure to be used in selection. Study 1 described the creation of an item bank and the selection of the 150 best-performing items. Study 2 described the development of a predictive machine-learning-based scoring algorithm for each trait and the validation of the image-based measure by measuring convergent validity with a validated, questionnaire-based measure and testing for potential adverse impact. 

### 4.1. Model Performance 

The findings of this study provide preliminary evidence that all five personality traits can be accurately measured via image choices in a similar way to traditional measures of personality. Models were trained on 70% of the data then cross-validated with the remaining test data to assess generalizability. The model’s accuracy was assessed by correlating scores on the image- and questionnaire-based measures. Across all five traits, correlations were strong for both the training and test data, indicating good model accuracy and generalizability to unseen data (Jacobucci et al. 2016). The convergent validity between the image- and questionnaire-based measures, determined by correlations for the test set, ranged from 0.60 for agreeableness to 0.78 for extraversion. These values exceed those reported by previous non-verbal personality measures; correlations between the Nonverbal Personality Questionnaire and the NEO-FFI (McCrae and Costa 1985) ranged from 0.45 for emotional stability to 0.59 for agreeableness (Paunonen et al. 2001). The convergent validity range for this measure is comparable to the convergent validity between different questionnaire-based measures of personality, with correlations between scores on the IPIP (Goldberg 1992) and NEO-FFI (McCrae and Costa 1985), ranging from 0.50 for agreeableness to 0.76 for emotional stability (Lim and Ployhart 2006). However, since some discriminate correlations exceeded the values for convergent ones or were of a similar magnitude, this limits the conclusions that can be made about the validity of the assessment and highlights the need for further investigation. 

### 4.2. Methodological Considerations

While this assessment performed well in terms of accuracy and convergent validity, the sample size was small relative to other studies that describe predictive personality measures (Bachrach et al. 2012; Kosinski et al. 2013; Leutner et al. 2017; Schwartz et al. 2013). Although this could have implications for the model’s performance (Raudys and Jain 1991; Vabalas et al. 2019), the use of a train/test split is likely to have reduced the potential for the sample size to negatively impact the models (Vabalas et al. 2019). Additionally, although the size of the sample was relatively small, the range of personality scores was large, suggesting that the sample represents a range of personalities. Nevertheless, a larger sample size would likely have been beneficial, with the potential for creating more robust models. 

Furthermore, while the majority of items were mapped onto the trait that they were designed to measure in study 1, some were not, suggesting that it is difficult to perfectly align text- and image-based measures. This may be because image-based measures rely on personal interpretation of meaning which may vary between people. Despite this, all items were included as predictors for each model to examine which items were retained in the models and whether they aligned with the mapping. 

### 4.3. Future Validation

Before this assessment can be used in practice, further validation is needed to more robustly explore bias, generalizability, and how comparable this assessment is to other questionnaire-based measures. We suggest the following:

- User experience: To better understand how respondents engage with the measure, future studies could examine user experience, including how engaging respondents found the measure to be. It could also investigate whether the meaning assigned to the items by the team of designers converges with that of the respondents by asking a sample of respondents to assign their own adjectives to the items. This would allow further refinements to be made to the measure which may strengthen its performance;
- The potential for shorter measures: Since only a small number of predictors were retained by each model and there was some crossover in the predictors retained by the models, future studies should investigate how shorter versions of the assessment could take advantage of this by examining how effective different item combinations are at measuring the traits. This would result in even shorter testing times for candidates, reducing the time it takes to complete the overall battery of selection assessments;
- Bias and transparency: Group differences in scores do not always indicate bias and can instead be reflective of genuine differences in latent levels of traits for different subgroups (Society for Industrial and Organizational Psychology 2018). However, even when group differences in scores are not due to differences in ability, they do not always lead to adverse impact, especially when the analysis is based on a small sample (EEOC et al. 1978). Therefore, further validation is needed with a larger sample to more robustly determine whether the reported group differences could result in adverse impact, particularly since the importance of transparency and fairness in the algorithms used in hiring is increasingly a point of concern (Kazim et al. 2021; Raghavan et al. 2020);
- Mitigating bias: While the potential for adverse impact from this assessment echoes concerns about the fairness of conventional selection assessments (Hough et al. 2001), adverse impact associated with algorithmic recruitment processes can be mitigated by removing the items associated with group differences and updating the algorithms (HireVue 2019; Pymetrics 2021), unlike with traditional assessments that use a standard scoring key. Further research exploring the potential for mitigating group differences in the algorithms used by this assessment is needed, particularly since there is evidence of measurement bias in the questionnaire-based measure used to construct and validate the algorithms. Follow-up studies are, therefore, required to investigate the best way to mitigate this;
- Generalizability: The samples used in this study may be limited if they did not represent a diverse group of respondents. For example, data referring to the occupation of respondents were not collected, meaning the generalizability of the findings could be limited to a particular industry if respondents are from a similar background. To address this, a future study should recruit an additional sample from another source such as MTurk to validate the algorithm in a different population of respondents who are likely to have different attributes to those in the current samples;
- Cultural appropriateness: As only English-speaking respondents were included in this study, a variation in the interpretation of the items was not investigated across multiple cultures or languages. Whilst it is suggested that non-verbal assessments can be applied to any language without redevelopment (Paunonen et al. 1990), it is still important to ascertain whether the images included in this assessment are appropriate in other cultures. The findings of this study indicate that there are potential differences in the interpretation of the images for different subgroups, with convergence being null on some traits for Asian and Black respondents. Therefore, future studies should take a cross-cultural approach to investigate the performance of the measure in different cultures and ethnicities;
- Score inflation: Job application contexts have higher stakes as they can affect career-related opportunities (Stobart and Eggen 2012). Since there is evidence for the inflation of personality scores in high-stakes contexts (Arthur et al. 2010), a future study could investigate score inflation on this novel assessment in a high-stakes context. The forced-choice image-based format might decrease candidates’ ability to fake their responses compared to questionnaire based tests;
- Measure reliability: Respondents only took the measure once, meaning that response stability and consistency (test-retest reliability) could not be examined. Thus, it is not known whether respondents are likely to make the same image choices and therefore have similar personality scores each time they take the assessment. Further validation is needed to determine the test-retest reliability of this assessment;
- Measure validity: Additionally, further investigation is needed into other forms of validity, including internal validity since items mapped to multiple different traits were used in the models to predict each Big Five trait;
- Multitrait-multimethod approach: To better compare this measure to other traditional assessments, a future study using a multitrait-multimethod approach would provide insight into how the measure performs in terms of convergent and divergent validity with multiple other assessments (Dumenci 2000). Such an approach could also investigate whether user experience is greater for the image-based assessment as compared to traditional assessments, as has been previously indicated (Georgiou and Nikolaou 2020; Leutner et al. 2020).

### 4.4. Implications 

The reported study contributed towards addressing the lack of validated gamified assessments of personality, particularly assessments using an image-based format. Since GBAs are reportedly more engaging (Lieberoth 2015), elicit greater satisfaction (Downes-Le Guin et al. 2012; Leutner et al. 2020), and have shorter testing times (Atkins et al. 2014; Leutner et al. 2020) than traditional assessments, this measure could offer a more positive experience for applicants than traditional psychometric assessments. As applicants who view the selection process of an organization more positively reportedly have more positive perceptions of the employer and are more likely to accept a job offer (Hausknecht et al. 2004), this has implications for businesses as attractive selection methods can avoid offer rejections from talented candidates. The findings of this validation study provide preliminary evidence that all five personality traits can be measured through image choices, with the novel assessment showing promise for use in selection following further validation. 

## 5. Conclusions

Overall, this study found that image-based measures may be a valid and fair alternative form of assessment that could be used in place of traditional assessments using Likert scales. Using predictive scoring algorithms, the image-based assessment of personality described in this study demonstrates convergent validity with a validated, questionnaire-based measure comparable with the convergence between other questionnaire-based personality measures, suggesting that the reported assessment measures the Big Five traits in a similar way to traditional measures. Furthermore, this study found that the image-based measure is largely free from group differences which could potentially lead to adverse impact; however, further studies are needed using larger samples to test this more robustly. The measure also needs to be further validated to assess test-retest reliability and score inflation, as well as in other languages and cultures. Further studies could also compare user experience for the image-based assessment and a questionnaire-based measure. These preliminary findings have positive implications for the use of this assessment in selection; however, there is scope for further validation before this measure can be used in practice. 

## Figures and Tables

**Table 1:** The descriptive statistics for the questionnaire- and image-based measures.

Trait	Mean	SD	Min	Max	Range	Skewness	Kurtosis
Questionnaire-based measure							
Openness	82.84	12.02	34.00	114.00	80.00	−0.32	−0.32
Conscientiousness	86.31	15.14	16.00	120.00	104.00	−0.54	−0.54
Extraversion	75.98	15.12	11.00	114.00	103.00	−0.30	−0.30
Agreeableness	90.32	13.64	13.00	119.00	106.00	−1.11	−1.11
Emotional stability	76.10	18.69	10.00	120.00	110.00	−0.27	−0.27
Image-based measure							
Openness	82.89	7.19	59.16	102.31	43.16	−0.05	0.03
Conscientiousness	86.79	10.68	52.17	110.84	58.67	−0.51	−0.12
Extraversion	76.07	11.77	44.20	101.97	57.77	−0.11	−0.65
Agreeableness	90.20	8.46	59.80	110.54	50.74	−0.54	0.51
Emotional stability	75.71	12.67	42.35	100.84	58.49	−0.37	−0.49

**Table 2:** A correlation matrix for the questionnaire-based measure (Sample 2; N = 431).

Trait	1	2	3	4	5
1. Openness	0.83				
2. Conscientiousness	0.12 *	0.91			
3. Extraversion	0.33 **	0.43 **	0.90		
4. Agreeableness	0.34 **	0.52 **	0.24 **	0.89	
5. Emotional stability	0.09	0.61 **	0.60 **	0.35 **	0.93

*Note. Diagonal values represent Cronbach’s alpha coefficient*. * *p* < 0.05. ** *p* < 0.001.

**Table 3:** The model performance for the image-based assessment.

Trait	Training (*n* = 323)			Test (*n* = 108)		
	r	R^2^	MSE	r	R^2^	MSE
Openness	0.77 **	0.56	65.63	0.71 **	0.50	64.46
Conscientiousness	0.82 **	0.66	82.62	0.70 **	0.47	97.26
Extraversion	0.86 **	0.74	61.23	0.78 **	0.61	82.28
Agreeableness	0.77 **	0.56	84.97	0.60 **	0.34	103.03
Emotional Stability	0.80 **	0.63	131.35	0.70 **	0.47	175.29

*Note. r* = Pearson’s correlation coefficients with the equivalent trait on the questionnaire-based assessment. ** *p* < 0.001.

**Table 4:** A multitrait-multimethod matrix investigating the convergent and discriminant validity of the image- and questionnaire-based measures (Test set of sample 2; *n* = 108).

Trait	1	2	3	4	5	6	7	8	9	10
Questionnaire-based										
1. Openness	1.00									
2. Conscientiousness	0.10	1.00								
3. Extraversion	0.29 **	0.35 **	1.00							
4. Agreeableness	0.31 **	0.50 **	0.12	1.00						
5. Emotional stability	0.01	0.63 **	0.54 **	0.30 **	1.00					
Image-based										
6. Openness	0.71 **	0.05	0.39 **	0.31 **	0.06	1.00				
7. Conscientiousness	−0.04	0.70 **	0.21 *	0.32 **	0.54 **	0.00	1.00			
8. Extraversion	0.26 **	0.30 **	0.78 **	0.12	0.52 **	0.48 **	0.32 **	1.00		
9. Agreeableness	0.18	0.42 **	0.13	0.60 **	0.23 *	0.46 **	0.54 **	0.22 *	1.00	
10. Emotional stability	0.08	0.51 **	0.57 **	0.21 *	0.70 **	0.19	0.69 **	0.71 **	0.38 **	1.00

* *p* < 0.05. ** *p* < 0.001.

**Table 5:** The adverse impact analysis for the image-based assessment based on the four-fifths rule, two standard deviations rule, and Cohen’s *d*.

Demographic	Group Size (N = 431)	Respondents Passing (*n*)	Respondents Passing (%)	Adverse Impact Ratio	Standard Deviations	Cohen’s *d*
Openness						
Gender						
Male	205	79	38.54	0.68	−3.67	0.36
Female	222	125	56.31	1.00	3.65	0.00
Age						
40 or older	75	32	42.67	1.00	0.98	0.00
Under 40	356	174	48.88	0.87	−0.98	0.12
Ethnicity						
White	209	106	50.72	0.95	1.18	0.05
Black	73	36	49.32	0.92	0.29	0.08
Asian	66	19	28.79	0.54	−3.36	0.52
Hispanic	56	30	53.57	1.00	0.93	0.00
Conscientiousness						
Gender						
Male	205	102	49.76	0.83	−1.89	0.20
Female	222	133	59.91	1.00	2.31	0.00
Age						
40 or older	75	44	58.67	0.91	−0.79	0.10
Under 40	356	191	53.65	1.00	0.79	0.00
Ethnicity						
White	209	114	54.55	0.80	0.01	0.23
Black	73	50	68.49	1.00	2.63	0.00
Asian	66	36	54.55	0.80	0.00	0.29
Hispanic	56	24	42.86	0.63	−1.88	0.53
Extraversion						
Gender						
Male	205	106	51.71	1.00	0.08	0.00
Female	222	115	51.8	1.00	0.13	0.00
Age						
40 or older	75	40	53.33	0.96	−0.35	0.04
Under 40	356	182	51.12	1.00	0.35	0.00
Ethnicity						
White	209	109	52.15	0.98	0.26	0.02
Black	73	39	53.42	1.00	0.36	0.00
Asian	66	33	50	0.94	−0.27	0.07
Hispanic	56	27	48.21	0.90	−0.53	0.10
Agreeableness						
Gender						
Male	205	91	44.39	0.68	−4.02	0.42
Female	222	144	64.86	1.00	4.44	0.00
Age						
40 or older	75	45	60	0.89	−1.05	0.13
Under 40	356	190	53.37	1.00	1.05	0.00
Ethnicity						
White	209	124	59.33	0.98	1.94	0.02
Black	73	44	60.27	1.00	1.08	0.00
Asian	66	29	43.94	0.73	−1.88	0.33
Hispanic	56	22	39.29	0.65	−2.46	0.43
Emotional Stability						
Gender						
Male	205	118	57.56	1.00	1.66	0.00
Female	222	111	50	0.87	−1.44	0.15
Age						
40 or older	75	39	52	1.00	0.26	0.00
Under 40	356	191	53.65	0.97	−0.26	0.03
Ethnicity						
White	209	109	52.15	0.86	−0.49	0.14
Black	73	42	57.53	0.95	0.78	0.06
Asian	66	40	60.61	1.00	1.28	0.00
Hispanic	56	27	48.21	0.80	−0.83	0.25

**Table 6:** The adverse impact analysis for the questionnaire-based assessment based on the four-fifths rule, two standard deviations rule, and Cohen’s *d*.

Demographic	Group Size (N = 431)	Respondents Passing (*n*)	Respondents Passing (%)	Adverse Impact Ratio	Standard Deviations	Cohen’s *d*
Openness						
Gender						
Male	205	93	45.37	0.77	−3.54	0.27
Female	222	130	58.56	1.00	3.75	0.00
Age						
40 or older	75	29	38.67	0.70	−1.87	0.34
Under 40	356	197	55.34	1.00	1.87	0.00
Ethnicity						
White	209	108	51.67	0.80	0.26	0.21
Black	73	41	56.16	0.87	0.10	0.17
Asian	66	23	34.85	0.54	−0.80	0.61
Hispanic	56	36	64.29	1.00	0.33	0.00
Conscientiousness						
Gender						
Male	205	95	46.34	0.80	−2.23	0.24
Female	222	129	58.11	1.00	2.63	0.00
Age						
40 or older	75	43	57.33	1.00	−1.02	0.00
Under 40	356	181	50.84	0.89	1.02	0.13
Ethnicity						
White	209	111	53.11	0.86	0.46	0.14
Black	73	45	61.64	1.00	1.81	0.00
Asian	66	33	50.00	0.81	−0.35	0.23
Hispanic	56	27	48.21	0.78	−0.60	0.27
Extraversion						
Gender						
Male	205	102	49.76	0.92	−0.79	0.09
Female	222	120	54.05	1.00	0.99	0.00
Age						
40 or older	75	39	52.00	1.00	−0.05	0.00
Under 40	356	184	51.69	0.99	0.05	0.01
Ethnicity						
White	209	106	50.72	0.96	−0.41	0.04
Black	73	38	52.05	0.98	0.06	0.02
Asian	66	35	53.03	1.00	0.23	0.00
Hispanic	56	28	50.00	0.94	−0.28	0.06
Agreeableness						
Gender						
Male	205	93	45.37	0.70	−3.82	0.40
Female	222	144	64.86	1.00	4.25	0.00
Age						
40 or older	75	48	64.00	1.00	−1.73	0.00
Under 40	356	189	53.09	0.83	1.73	0.22
Ethnicity						
White	209	140	66.99	1.00	4.86	0.00
Black	73	27	36.99	0.55	−3.39	0.50
Asian	66	26	39.39	0.59	−2.77	0.57
Hispanic	56	31	55.36	0.83	0.06	0.24
Emotional Stability						
Gender						
Male	205	125	60.98	1.00	3.75	0.00
Female	222	96	43.24	0.71	−3.54	0.36
Age						
40 or older	75	46	61.33	1.00	−1.87	0.00
Under 40	356	176	49.44	0.81	1.87	0.24
Ethnicity						
White	209	109	52.15	0.97	0.26	0.02
Black	73	38	52.05	0.97	0.10	0.03
Asian	66	31	46.97	0.88	−0.80	0.13
Hispanic	56	30	53.57	1.00	0.33	0.00

**Table 7:** The convergent correlations by subgroup. O, C, E, A, and ES refer to Openness, Conscientiousness, Extraversion, Agreeableness, and Emotional Stability, respectively.

Demographic	O	C	E	A	ES
Gender					
Male	0.65 **	0.76 **	0.80 **	0.69 **	0.72 **
Female	0.76 **	0.58 **	0.76 **	0.42 **	0.69 **
Age					
Under 40 years old	0.67 **	0.68 **	0.80 **	0.50 **	0.68 **
Age 40 or older	0.78 **	0.76 **	0.68 **	0.86 **	0.77 **
Ethnicity					
White	0.77 **	0.75 **	0.83 **	0.62 **	0.83 **
Black	0.69 **	−0.01	0.86 **	0.05	0.73 **
Asian	0.65 **	0.72 **	0.85 **	0.08	0.45
Hispanic	0.56 **	0.72 **	0.83 **	0.83 **	0.57 *

* *p* < 0.05. ** *p* < 0.001.

## Data Availability

Data was obtained from HireVue and is not publicly available.

## Supplementary Materials

The following supporting information can be downloaded at: https://www.mdpi.com/article/10.3390/jintelligence10010012/s1, Table S1. Cohen’s *d* values and Lasso coefficients for the 300 items (150 image pairs) retained in study 1. Table S2: Coefficients of the images retained by each scoring algorithm in study 2 and the trait they were designed to measure and mapped to in study 1.
