# Supplementary material for: Measuring Personality through Images: Validating a Forced-Choice Image-Based Assessment of the Big Five Personality Traits
Source: J Intell. 2022 Feb 7;10(1):12. doi: 10.3390/jintelligence10010012 (PMC8883940; doi:10.3390/jintelligence10010012)
Supplement: Supplementary file 1 [file jintelligence-10-00012-s001.zip › jintelligence-1532346-supplementary.pdf]

Table S1. Cohen’s *d* values and Lasso coefficients for the 300 items (150 image pairs) retained in study 1.

	N respondents selecting		Cohen's *d*					Image retained in model				
Mapped trait(s)	Image 1	Image 2	O	C	E	A	N	O	C	E	A	ES
O	183	249	-0.69	0.00	-0.17	-0.39	-0.13	1		1, 2		
A/ES	249	180	0.34	0.03	0.27	0.54	-0.30	1			1	
C/A	193	237	-0.21	0.31	-0.37	-0.27	0.32	1				
ES	189	240	-0.21	-0.17	-0.72	0.00	0.73	1				
O/C	179	252	0.48	-0.21	0.56	-0.21	-0.35	1				
O	104	325	-0.76	0.31	-0.33	-0.35	0.12	2				
O	325	105	0.83	-0.23	0.36	-0.25	-0.24	2				
O	209	222	-0.54	-0.37	0.22	0.06	0.10	2				
C/ES	144	284	-0.26	-0.20	-0.60	-0.24	0.57	2				
A/ES	242	189	-0.09	0.19	-0.43	-0.43	0.27	1, 2				
ES/C	243	188	0.16	-0.46	-0.33	-0.37	0.34	1, 2				
A	144	285	-0.14	-0.85	0.42	-1.03	-0.01		1		2	1
E	78	352	-0.29	-0.66	-0.94	0.27	0.50		1			
C	281	147	-0.04	1.51	0.27	0.04	-0.54		1			
O	314	116	0.57	0.33	-0.06	0.20	-0.41		1			
C	189	240	0.31	-0.91	-0.93	0.11	0.75		2	2	2	1
ES	263	166	0.30	0.30	1.07	0.18	-0.77		2			2
O/C	206	224	0.92	-0.42	-0.05	-0.13	0.24		2			2
E	131	297	-0.34	-0.30	-0.94	0.00	0.47		2			
C	320	111	-0.30	0.61	0.22	0.09	-0.65		2			
O/ES	175	256	0.34	-0.39	0.55	0.17	-0.56		2			
A	323	107	0.24	0.59	0.66	0.76	-0.24		1, 2	1, 2	1, 2	
ES	158	273	0.08	-0.51	-0.71	0.12	0.84		1, 2	1,2		1,2
C	267	165	0.10	0.81	0.49	-0.18	-0.59		1, 2			1,2

	N respondents selecting		Cohen's *d* (based on study one)					Image retained in model				
Mapped trait(s)	Image 1	Image 2	O	C	E	A	N	O	C	E	A	ES
C	273	158	0.07	0.77	-0.10	0.57	-0.06		1, 2			
C	132	299	0.14	-0.75	-0.01	-0.59	0.05		1, 2			
C	205	226	0.12	-0.86	-0.24	0.03	0.31		1, 2			
C	220	210	0.22	-0.74	0.41	-0.61	-0.05		1, 2			
E/C	176	255	0.19	-0.39	0.34	-0.18	-0.19		1, 2			
ES	180	248	-0.32	-0.38	-0.86	-0.07	0.76			1		2
ES/O	265	164	-0.47	-0.13	0.14	-0.15	0.25			1		
E	248	182	-0.15	-0.34	-1.00	0.15	0.43			1		
E	207	223	-0.02	0.14	1.19	0.27	-0.59			2		2
E	241	188	0.03	0.53	-0.85	0.51	0.11			2		2
C/ES	238	191	-0.12	-0.22	-0.95	-0.24	0.75			2		2
E	259	171	-0.38	-0.48	-1.27	-0.02	0.83			2		
E	243	187	-0.14	-0.28	-1.14	-0.13	0.57			2		
O/E	211	220	0.31	-0.21	-1.17	-0.31	0.44			2		
O/ES	243	188	-0.28	0.01	-0.76	0.25	0.61			1, 2		1,2
O	54	377	0.59	-0.26	-0.27	-1.77	0.51			1, 2		
C/E	299	132	-0.17	0.33	-0.72	0.27	-0.19			1, 2		
C/E	157	273	-0.01	-0.35	0.50	-0.62	-0.30			1, 2		
O/ES	202	229	0.43	0.05	0.53	-0.30	-0.49			1, 2		
E/C	141	289	-0.05	-0.22	0.72	-0.39	-0.27			1, 2		
O/ES	161	270	0.33	-0.02	1.35	-0.54	-0.93			1, 2		
E/O	174	257	-0.43	-0.02	0.97	0.09	-0.31			1, 2		
O	104	328	-0.78	-0.72	-0.39	-0.74	0.05				1	
O/E	222	207	0.71	0.24	-0.29	0.48	0.01				1	
O/A	91	339	0.25	0.07	-0.22	-0.71	0.22				1	
A	73	358	-0.53	-0.42	0.21	-1.36	-0.28				1	

	N respondents selecting		Cohen's *d* (based on study one)					Image retained in model				
Mapped trait(s)	Image 1	Image 2	O	C	E	A	N	O	C	E	A	ES
A	346	84	-0.04	0.68	-0.01	0.67	0.11				1	
A/C	239	191	0.24	-0.85	0.24	0.55	0.13				1	
E/ES	155	277	0.18	0.00	0.73	-0.19	-1.28				1	
C	364	66	0.27	1.34	0.25	0.39	-0.19				2	
E/A	143	287	-0.17	0.14	0.47	-0.65	-0.49				2	
E/C	172	257	-0.18	-0.73	0.47	-0.40	0.06				2	
O	112	318	-0.54	-0.04	-0.43	-0.48	0.34				1, 2	
A	344	86	0.35	-0.01	0.02	1.09	0.01				1, 2	
A/E	343	88	-0.59	-0.07	-0.39	0.82	0.07				1, 2	
O/ES	140	291	0.34	0.26	-0.28	-0.12	-0.36				1, 2	
A/C	204	225	0.38	-0.59	-0.20	0.21	0.49					1
ES	164	265	-0.11	-0.49	-0.61	0.06	1.11					2
ES	164	267	-0.23	-0.36	-0.76	0.21	0.73					1,2
ES	278	153	-0.36	-0.39	-0.76	0.04	0.69					1,2
A	289	143	0.13	-0.21	-0.37	-0.52	0.29					1,2
ES	266	165	-0.03	0.16	0.43	0.00	-1.01					1,2
E/O	285	145	-0.54	0.03	0.44	0.02	-0.37					1,2
E/A	194	236	-0.13	0.18	0.60	-0.36	-0.79					1,2
A/O	281	149	-0.40	0.28	0.10	0.39	0.16					1,2
ES	328	104	0.19	-0.02	-0.49	0.31	0.71					
A	328	101	0.17	0.71	-0.33	0.63	0.11					
A	342	89	0.57	0.59	0.70	0.58	-0.72					
A	71	359	-0.24	-0.79	0.48	-0.61	-0.11					
A	364	65	-0.22	0.31	-0.13	0.97	-0.19					
A	359	70	0.34	0.68	0.48	0.53	-0.71					
E/ES	172	257	-0.05	-0.04	0.47	0.13	-0.40					

	N respondents selecting		Cohen's *d* (based on study one)					Image retained in model				
Mapped trait(s)	Image 1	Image 2	O	C	E	A	N	O	C	E	A	ES
E/ES	253	179	-0.22	0.06	-0.61	0.01	0.61					
E/A	130	301	-0.13	-0.17	0.54	-0.41	-0.50					
ES/C	90	341	-0.20	-0.53	-0.81	-0.63	0.76					
E/ES	262	169	-0.35	0.16	0.73	0.23	-0.44					
E/O	224	208	-0.62	0.02	0.49	-0.06	0.25					
A/ES	177	253	0.16	0.00	0.61	0.28	-0.43					
O/A	198	233	0.39	-0.16	-0.17	-0.39	0.21					
O/E	167	263	0.64	-0.08	-0.24	-0.05	0.14					
A/ES	222	209	0.24	0.39	-0.29	0.24	-0.58					
A/ES	182	249	-0.02	-0.37	-0.35	-0.49	0.24					
C/ES	171	258	0.03	0.49	0.23	0.13	-0.37					
ES/C	254	177	-0.10	-0.31	-0.43	0.09	0.45					
A/C	173	258	0.29	-0.45	0.15	0.22	-0.06					
O	47	382	-0.57	-0.54	-0.69	-0.27	0.51					
C	281	148	0.02	1.06	0.47	0.19	-0.62					
A	178	253	0.21	-0.30	0.30	-0.55	-0.50					
O	108	323	-0.51	0.35	-0.25	0.24	-0.18					
C	98	333	0.03	-0.82	-0.14	-0.40	0.03					
O	109	321	0.54	0.10	0.21	-0.04	-0.13					
C	193	239	0.42	0.72	-0.04	0.38	0.23					
E	225	206	-0.13	-0.38	-1.12	-0.43	0.51					
E	312	120	-0.27	-0.13	-0.87	0.04	0.29					
E	292	138	0.19	0.39	1.08	0.14	-0.83					
E	226	202	0.20	-0.09	1.00	0.08	-0.49					
O	264	166	-0.48	-0.35	-0.66	0.02	0.49					
E	197	233	0.11	-0.29	-1.13	-0.12	0.67					

	N respondents selecting		Cohen's *d* (based on study one)					Image retained in model				
Mapped trait(s)	Image 1	Image 2	O	C	E	A	N	O	C	E	A	ES
C	354	75	-0.44	0.96	-0.81	0.52	0.04					
E	224	208	0.17	-0.11	-0.94	0.20	0.37					
C	242	190	-0.04	0.78	0.48	0.36	-0.50					
O	71	359	-1.05	-0.15	-0.51	0.24	0.22					
E	342	86	-0.51	0.18	-1.03	0.49	0.45					
ES	284	146	0.03	0.35	0.53	0.04	-0.83					
ES	109	322	-0.41	-0.62	-1.04	-0.28	0.80					
ES	165	266	-0.19	-0.65	-0.94	0.04	1.17					
C	301	129	0.19	0.73	0.25	0.46	-0.39					
A	180	250	-0.45	-0.40	0.05	-0.70	-0.01					
C/A	280	150	0.51	-0.31	-0.22	0.23	0.44					
C/ES	302	128	0.03	-0.41	-0.65	0.06	0.56					
C/E	268	163	0.08	0.51	-0.28	-0.09	-0.19					
E/A	162	269	0.13	-0.03	0.30	-0.64	-0.26					
A/ES	281	150	0.53	0.22	0.59	0.47	-0.34					
ES/O	190	241	0.37	-0.37	0.00	0.35	-0.58					
E/A	252	179	-0.06	0.12	-0.89	0.26	0.12					
O/E	254	175	-0.35	0.06	0.94	0.27	-0.24					
C/O	269	161	-0.30	0.34	-0.40	-0.12	-0.04					
C/A	202	230	-0.14	0.39	0.32	-0.78	-0.36					
C/ES	222	209	-0.05	-0.38	-0.74	-0.10	0.40					
A/E	207	224	-0.34	0.20	0.24	-0.82	-0.22					
C/E	333	98	0.08	0.95	-0.48	0.69	-0.01					
O/ES	331	100	-0.67	0.36	-0.84	-0.05	0.40					
C/O	301	129	-0.58	0.69	-0.48	-0.14	0.14					
C/ES	195	235	-0.21	-0.36	-0.48	0.18	0.90					

	N respondents selecting		Cohen's *d* (based on study one)					Image retained in model				
Mapped trait(s)	Image 1	Image 2	O	C	E	A	N	O	C	E	A	ES
A/E	269	161	0.38	0.15	-0.91	0.34	0.41					
O/A	116	313	0.37	-0.19	0.32	-0.68	-0.31					
O/A	144	287	0.28	-0.11	0.84	-0.82	-0.36					
E/ES	205	225	0.32	0.18	0.58	0.05	-0.49					
E/ES	182	248	-0.20	-0.14	0.94	-0.16	-0.69					
A/ES	312	119	0.18	0.25	0.57	1.02	-0.47					
E/C	196	235	-0.01	-0.51	0.53	0.05	-0.35					
E/O	254	176	-0.65	0.00	0.83	-0.45	-0.43					
E/ES	207	224	0.09	-0.19	0.83	-0.14	-0.35					
E/ES	150	281	0.51	0.16	0.61	0.16	-0.81					
C/A	290	141	-0.04	-0.22	-0.28	0.26	0.29					
ES/A	204	224	0.17	0.06	-0.56	-0.42	0.42					
O/A	202	230	0.30	0.00	0.19	-0.44	-0.22					
ES/A	164	267	0.23	-0.17	-0.52	-0.55	0.28					
O/A	142	290	0.75	-0.24	0.33	-0.29	-0.34					
O/A	196	234	0.35	-0.02	0.33	-0.82	-0.29					
O/C	184	245	0.42	-0.73	-0.26	-0.37	0.61					
A/C	175	254	0.27	-0.71	-0.29	-0.25	0.21					
O/C	209	222	0.91	-0.26	0.12	0.07	-0.31					
A/E	265	166	0.36	-0.17	-0.48	0.40	0.21					
O/E	230	202	0.62	0.13	-0.38	0.58	0.36					
ES/E	232	199	0.04	0.05	-1.05	-0.29	0.29					

Table S2: Coefficients of the images retained by each scoring algorithm in study 2 and the trait they were designed to measure and mapped to in study 1.

Designed to measure	Mapped to	Lasso coefficient
**Conscientiousness**		
Conscientiousness: orderliness	C	-0.0214764
Openness: artistic interests	O	-0.124441
Openness: imagination	O	0.828066
Openness: imagination	O	-4.11045
Openness: intellect	O	0.170194
Emotional stability: self-consciousness	ES	-0.322042
Agreeableness: trust	A	0.554836
Conscientiousness: dutifulness	ES	-1.12284
Agreeableness: trust	A	0.237
Emotional stability: self-consciousness	ES	1.59597
Extraversion: excitement-seeking	O	0.949553
Conscientiousness: achievement-striving	ES	-0.0994327
Openness: intellect	C	0.568391
**Openness**		
Conscientiousness: orderliness	C	4.59555
Conscientiousness: orderliness	C	2.78319
Conscientiousness: self-discipline	C	2.38049
Openness: artistic interests	O	2.04351
Conscientiousness: self-efficacy	C	0.960278
Emotional stability: vulnerability	C	0.534107
Emotional stability: vulnerability	ES	0.519069
Agreeableness: sympathy	A	0.470262
Conscientiousness: orderliness	ES	0.41159
Agreeableness: altruism	E	0.357729
Conscientiousness: achievement-striving	C	0.180862

Conscientiousness: self-efficacy	C	0.180556
Conscientiousness: achievement-striving	E	0.0312252
Conscientiousness: orderliness	C	0.0208352
Agreeableness: sympathy	A	-0.0000449482
Emotional stability: vulnerability	ES	-0.0000529599
Conscientiousness: self-discipline	C	-0.00313537
Agreeableness: modesty	E	-0.141738
Openness: adventurousness	C	-0.201648
Conscientiousness: achievement-striving	C	-0.27121
Emotional stability: self-consciousness	ES	-0.298578
Extraversion: gregariousness	A	-0.628303
Conscientiousness: self-discipline	C	-1.54706
Conscientiousness: orderliness	C	-2.09994
Conscientiousness: self-efficacy	C	-2.13473
Emotional stability: vulnerability	C	-3.39599
**Extraversion**		
Emotional stability: self-consciousness	E	4.14099
Conscientiousness: achievement- striving	O	3.0337
Extraversion: cheerfulness	E	2.54409
Agreeableness: altruism	S	2.18124
Extraversion: cheerfulness	O	1.94808
Agreeableness: sympathy	A	1.57287
Extraversion: excitement-seeking	E	1.10023
Extraversion: assertiveness	S	1.04607
Conscientiousness: orderliness	C	0.880609
Extraversion: friendliness	O	0.792065
Extraversion: friendliness	C	0.60086
Conscientiousness: cautiousness	E	0.523434
Extraversion: friendliness	O	0.406253

Conscientiousness: achievement-striving	C	0.352042
Emotional stability: vulnerability	S	0.141487
Openness: artistic interests	O	0.11439
Emotional stability: self-consciousness	E	0.0905166
Extraversion: friendliness	O	0.00120838
Emotional stability: vulnerability	S	-0.000172458
Agreeableness: modesty	E	-0.000326526
Extraversion: cheerfulness	O	-0.0007981
Emotional stability: vulnerability	C	-0.172335
Agreeableness: sympathy	A	-0.285091
Openness: intellect	E	-0.300405
Extraversion: friendliness	E	-0.325052
Conscientiousness: cautiousness	O	-0.616223
Emotional stability: immoderation	S	-0.698025
Emotional stability: self-consciousness	S	-0.812753
Conscientiousness: cautiousness	O	-1.55058
Openness: imagination	E	-1.81113
Extraversion: friendliness	E	-2.14174
Openness: artistic interests	O	-2.40557
**Agreeableness**		
Agreeableness: trust	O	1.96295
Extraversion: assertiveness	A	1.6102
Agreeableness: altruism	A	1.5622
Conscientiousness: self-discipline	C	1.33769
Agreeableness: sympathy	A	1.15986
Extraversion: gregariousness	A	1.09962
Conscientiousness: orderliness	C	0.559115
Agreeableness: cooperation	O	0.403749
Agreeableness: trust	A	0.356484

Agreeableness: morality	A	0.313762
Agreeableness: sympathy	A	0.067053
Agreeableness: cooperation	A	0.018382
Emotional stability: self-consciousness	ES	0.015517
Extraversion: assertiveness	E	-0.00476
Agreeableness: sympathy	A	-0.00508
Agreeableness: trust	O	-0.01396
Agreeableness: altruism	O	-0.24923
Openness: adventurousness	O	-0.24977
Agreeableness: morality	C	-0.41997
Conscientiousness: dutifulness	A	-0.85676
Openness: emotionality	E	-1.5204
Agreeableness: cooperation	A	-1.66305
Conscientiousness: achievement-striving	O	-2.46459
**Emotional Stability**		
Emotional stability: vulnerability	ES	5.7346
Emotional stability: self-consciousness	ES	3.45296
Agreeableness: altruism	ES	2.99479
Emotional stability: vulnerability	ES	2.12548
Emotional stability: vulnerability	ES	1.5408
Agreeableness: cooperation	A	1.4023
Emotional stability: self-consciousness	E	1.36492
Extraversion: cheerfulness	E	0.548057
Conscientiousness: self-efficacy	C	0.500456
Extraversion: assertiveness	ES	0.330107
Conscientiousness: cautiousness	ES	0.210182
Emotional stability: vulnerability	C	0.124217
Extraversion: gregariousness	A	0.118446
Agreeableness: modesty	ES	0.018917

Extraversion: activity level	E	0.002031
Conscientiousness: cautiousness	O	-0.00343
Emotional stability: vulnerability	ES	-0.00728
Extraversion: gregariousness	A	-0.03076
Extraversion: gregariousness	A	-0.21115
Conscientiousness: cautiousness	ES	-0.22568
Openness: adventurousness	O	-0.52122
Emotional stability: vulnerability	ES	-0.57619
Extraversion: friendliness	E	-0.65542
Openness: emotionality	A	-0.95606
Emotional stability: self-consciousness	ES	-1.20979
Agreeableness: modesty	ES	-1.29436
Openness: artistic interests	O	-1.45664
Openness: emotionality	A	-1.95982
Conscientiousness: orderliness	C	-2.34316
Emotional stability: vulnerability	C	-3.24497
